# Identification of functional *cis*-regulatory elements by sequential enrichment from a randomized synthetic DNA library

**DOI:** 10.1186/1471-2229-13-164

**Published:** 2013-10-18

**Authors:** Mario Roccaro, Nahal Ahmadinejad, Thomas Colby, Imre E Somssich

**Affiliations:** 1 Department of Plant Microbe Interaction, Max Planck Institute for Plant Breeding Research, Carl-von-Linne-Weg 10, Cologne 50829, Germany; 2 Mass Spectrometry Group, Max Planck Institute for Plant Breeding Research, Carl-von-Linne-Weg 10, Cologne 50829, Germany; 3 Current address: INRES - Crop Bioinformatics, Universität Bonn, Katzenburgweg 2, Bonn 53115, Germany

**Keywords:** Chromatin immunoprecipitation (ChIP), Motif discovery, Plant protoplasts, Pol II CTD phosphorylation, Synthetic DNA elements

## Abstract

**Background:**

The identification of endogenous *cis*-regulatory DNA elements (CREs) responsive to endogenous and environmental cues is important for studying gene regulation and for biotechnological applications but is labor and time intensive. Alternatively, by taking a synthetic biology approach small specific DNA binding sites tailored to the needs of the scientist can be generated and rapidly identified.

**Results:**

Here we report a novel approach to identify stimulus-responsive synthetic CREs (SynCREs) from an unbiased random synthetic element (SynE) library. Functional SynCREs were isolated by screening the SynE libray for elements mediating transcriptional activity in plant protoplasts. Responsive elements were chromatin immunoprecipitated by targeting the active Ser-5 phosphorylated RNA polymerase II CTD (Pol II ChIP). Using sequential enrichment, deep sequencing and a bioinformatics pipeline, candidate responsive SynCREs were identified within a pool of constitutively active DNA elements and further validated. These included *bonafide* biotic/abiotic stress-responsive motifs along with novel SynCREs. We tested several SynCREs in *Arabidopsis* and confirmed their response to biotic stimuli.

**Conclusions:**

Successful isolation of synthetic stress-responsive elements from our screen illustrates the power of the described methodology. This approach can be applied to any transfectable eukaryotic system since it exploits a universal feature of the eukaryotic Pol II.

## Background

The availability of SynCREs that directly control gene expression in diverse cell types and upon environmental cues would prove invaluable to define signaling pathways, to isolate novel mutants via targeted genetics, and to engineer crop species for improved stress tolerance [1]. SynCREs offer several advantages over native promoters for bioengineering purposes. Native promoters often contain multiple CREs that can drastically modulate promoter strength in a positive or negative manner depending on the cellular context, reflecting the complexity of transcriptional regulation exerted by *trans*-acting factors. Moreover, the presence of elements mediating tissue- or hormone-specific and/or developmental expression in native promoters may limit their versatility. In contrast, a single or tandem version of a SynCRE normally reduces the overall transcriptional complexity [2-4]. A synthetic element can have a more defined spatial and temporal expression pattern suitable for driving transgene expression in a more tightly regulated fashion [5,6]. Delineation of in vitro CREs is laborious and requires extensive functional promoter dissections. *In silico* analyses are equally difficult. Despite sophisticated algorithms developed to search for motifs [7-10], subsequent *in vivo* functional validation of such elements is nonetheless required [11,12].

Synthetic DNA sequences have been successfully used as an unbiased source of CREs in animal cell lines employing a retroviral vector and fluorescent cell sorting [13,14]. Although plant cell lines also represent a valuable tool to study gene expression [15-17], they are often comprised of small cell aggregates thereby preventing sorting of individual cells. Also, their thick cell walls preclude the high transformation efficiencies necessary to screen complex SynE libraries. In contrast, parsley protoplasts have proven to be highly suitable for expression studies as they retain their responsiveness to environmental stimuli such as UV light and pathogen-derived elicitors [11,18]. Nevertheless, transfection efficiencies of plant protoplasts remain several orders of magnitude lower compared to virus-mediated transduction of animal cells.

In this study, we have devised a strategy to overcome some of the limitations associated with plant cell lines that allows us to use complex random SynE libraries and to isolate SynCREs capable of maintaining or enhancing transcriptional activity *in planta* upon elicitation.

## Results and discussion

**Figure 1:**
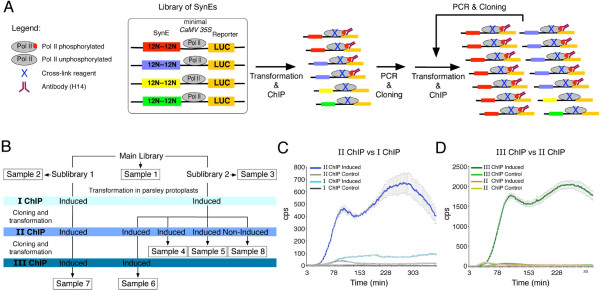
**Identification of synthetic *cis*-regulatory DNA elements (SynCREs) via Pol II ChIP and validation. (A)** Synthetic DNA element (SynEs) library was cloned into a binary vector upstream of the *CaMV 35S*-derived TATA-box to drive expression of the luciferase reporter gene (*LUC*), and transfected into parsley protoplasts. Pol II phosphorylation at Ser-5 is indicated. DNA-Pol II complexes were formaldehyde cross-linked and the SynEs mediating *LUC* expression were immunoprecipitated with an antibody (H14; indicated in red) specific to Ser-5 phosphorylated Pol II. The first chromatin immunoprecipitated SynEs (ChIP) were specifically PCR amplified and re-cloned to construct a subsequent library, which was used for a second transfection round and ChIP. By repeating these steps, an additional enrichment for SynEs mediating *LUC* expression was obtained. **(B)** Scheme for SynEs library screening, ChIP, and sample treatments. Three Pol II ChIP rounds were performed on transfected parsley protoplasts untreated or induced by Pep25, to enrich for responsive SynCREs. Eight samples were sequenced (boxed) comprising the main library, two sub-libraries and six samples. **(C)** Comparison of LUC activities in parsley protoplasts between SynEs contained in a first round ChIP (I ChIP) sample and in a subsequent ChIP-derived sample (II ChIP), either untreated (control) or Pep25 induced. The II ChIP sample showed higher LUC activity than the I ChIP sample. Both samples showed increased luciferase activities under inducing conditions compared to control. LUC activity was measured as counts per second (cps) of photon emission using the TopCount®. **(D)** Comparison of LUC activities between SynEs contained in a II ChIP sample and following a third ChIP (III ChIP) round, either under untreated or Pep25-induced conditions. The III ChIP sample showed higher LUC activity than the II ChIP sample. Increase of LUC activity observed in samples after each subsequent round of ChIP indicates enrichment for responsive SynCREs.

Our approach to identify stimulus-responsive CREs is based on the Pol II ChIP technique, targeted to the Ser-5 phosphorylated Pol II carboxy-terminal domain (CTD) [19-22]. Ser-5 phosphorylation occurs at promoter-proximal regions, is directly proportional to transcriptional activity, and correlates with the release of Pol II from the pre-initiation complex to initiate transcription [19,23-25]. Therefore, Pol-II ChIP enables the capture and enrichment of promoter fragments containing SynCREs actively supporting transcription within libraries transformed into cells or protoplasts (Figure 1). In this study, parsley protoplasts were stimulated using Pep25, a *Phytophthora sojae*-derived peptide that triggers plant immune responses [26].

The SynEs carried a tandem of two 12 randomized nucleotides (12 N) separated by a 20 base pair spacer sequence (Additional file 1: Figures S1, S2). This topology was adopted to link CREs often not associated in native promoters. Such composites can enhance transcriptional activity [13,27]. The SynE cassettes were cloned upstream of the minimal (-46 to +8 TATA box) *CaMV 35S* promoter driving the expression of a luciferase reporter gene (*LUC*) to construct a main library of randomized elements (Additional file 1: Figure S1). Two sub-libraries (Sample 2 and Sample 3) each comprised of about 1 × 10^6^ recombinant synthetic elements originating from the main library were transformed into parsley protoplasts and subjected to several subsequent rounds of Pol II ChIP (Figure 1a,b). The library was screened by monitoring *LUC* transcriptional activity *in vivo* for several hundreds of samples in parallel using a TopCount machine [28]. For the Pol II ChIP we utilized a commercially available monoclonal antibody (H14) recognizing the Ser-5 phosphorylated CTD of Pol II previously used successfully in Arabidopsis [24,29]. We verified the specificity of the antibody for four plant species including parsley (Additional file 1: Figure S3).

SynCREs immunoprecipitated from Pep25 elicited cells in a first ChIP round should represent a pool of active SynEs that are partly enriched for a set of SynEs mediating Pep25-dependent expression with respect to the original library. By comparing the reporter gene activities of cells transformed with the primary library with that of a subsequent library of SynEs, it was possible to draw conclusions regarding enrichment for SynCREs (Figure 1c, d).

Luciferase activity measurements confirmed that the two sub-libraries contained functional SynCREs (Additional file 1: Figure S4). The fact that the control parsley protoplasts initially showed overall higher *LUC* transcriptional activity following the first ChIP round compared to the Pep25-induced cells (Additional file 1: Figure S4a) is consistent with previous observations that Pep25 represses growth and overall metabolism of parsley cells during activation of selective defense responses [30,31]. Formaldehyde cross-linking was performed 3 hours post Pep25 treatment of the cells. The DNA fragments harboring the enriched set of SynEs of the first ChIP (I ChIP) were re-cloned and subjected to a second ChIP (II ChIP) (Figure 1). Hence, as expected, subsequent rounds of ChIP resulted in an enrichment of SynCREs within the pool of immunopecipitated SynEs mediating expression and/or responsiveness to Pep25 (Figure 1c, d).

Overall, eight samples were subjected to deep sequencing; the main library (Sample 1), two sub-libraries (Sample 2, 3) and six samples at different rounds of ChIP (Samples 4–8, Figure 1b). Barcoding was employed to discriminate between samples (Additional file 1: Figure S5). A bioinformatics pipeline was developed for sequence analysis data and to identify candidate responsive SynCREs (Additional file 1: Figure S6). Reads were filtered using the designed sequence pattern in order to recognize and exclude sequencing errors (see Methods). A non-redundant set of reads was extracted for each sample and showed that the libraries (Samples 1–3) contain >90% unique sequences thereby confirming their random synthetic oligonucleotide compositions (Table 1). In contrast, a drastic reduction of unique sequences was observed in the other samples (23%-33%; Samples 4–8, Table 1). This reduction in complexity is consistent with enrichment for selective SynEs during ChIP.

**Table 1:** Summary of sequences obtained

				**Non-redundant data set**		**Clustering**	
	**Samples**	**N**	N filtered*	**N° non-redundant**	**% unique**	**N°**	**% clustered**
		**sequences**	**sequences**	**sequences**	**sequences**	**clusters**	**sequences**
**Libraries**	**Sample 1**	**958.534**	**646.511**	**645.835**	**99,9**	**645.835**	**0**
	**Sample 2**	**392.428**	**259.149**	**234.498**	**90,5**	**231.838**	**1,1**
	**Sample 3**	**370.017**	**248.009**	**227.334**	**91,7**	**225.081**	**0,9**
**II ChIP**	**Sample 4**	**481.873**	**299.152**	**82.834**	**27,7**	**44.055**	**46,8**
	**Sample 5**	**435.942**	**280.944**	**84.627**	**30,1**	**47.345**	**44,1**
	**Sample 8**	**308.053**	**201.329**	**66.654**	**33,1**	**38.251**	**42,6**
**III ChIP**	**Sample 6**	**510.785**	**312.151**	**71.701**	**22,9**	**39.151**	**45,4**
	**Sample 7**	**234.235**	**159.607**	**42.234**	**26,5**	**22.758**	**46,1**

The number (N°) of total sequences, the number of filtered sequences, the non-redundant sequence data set and the clustering sequence data obtained for the main- (Sample 1) and the two sub-libraries (Samples 2 +3), and following two rounds (II ChIP) or three rounds (III ChIP) of chromatin immunoprecipitation for the indicated samples. Asterisk denotes the number of filtered sequences that exactly matched the experimentally designed sequence pattern.

To further reduce complexity, we performed a clustering analysis using the program FreClu [32]. This analysis revealed that the library samples consisted of a large number of individual sequences, which could not be clustered. In contrast, the ChIP samples showed clustering above 40% (Table 1), revealing an enrichment of similar sequences indicative of SynE selection during the ChIP procedure. A sequence with the highest frequency within a cluster identified the representative SynE. By cluster comparisons we identified common representative SynEs among ChIP samples associated with the same enrichment stage (Additional file 1: Figure S7, Additional files 2 and 3). The samples derived from the third round of ChIP correlated with a reduction in the number of common clusters (Additional file 1: Figure S7b). One aspect of the selection system (Pol II ChIP) that would support a diversified enrichment is represented by the relatively low transformation efficiency of parsley protoplasts (10^4^-10^5^) compared with the number of SynEs. Thus, each protoplast transformation with ≈ 1×10^6^ synthetic elements should be viewed as a nearly independent experiment, and parallel ChIP experiments using the same sub-library will not necessarily isolate a highly overlapping set of SynEs. In addition, exhaustive deep sequencing of the samples would be required to fully reveal the extent of sequence overlap.

To uncover DNA motifs within the chromatin immunoprecipitated SynEs we applied different approaches. The frequencies of all possible sub-sequences (permutation) of 5, 6, and 7 bases long were calculated in each immunoprecipitated sample and compared to their frequencies in the sub-library from which the sample originated. The sub-sequence frequencies in the sub-library represented a randomized control set. A high frequency ratio between the sample and the sub-library for a particular sub-sequence indicated its potential functional importance within the SynEs, making it a candidate motif for further validation (Additional files 4, 5 and 6). Additionally, we analyzed the correlation of left and right 12 N sequence combinations. We found that individual 12 N-left or 12 N-right sequences of the synthetic element can be coupled to diverse 12 N-right or 12 N-left sequences, respectively. This could be due to the ability of a left- or right 12 N to support transcription in combination with different highly represented motifs embedded within the combined 12 N respective sequences. For each left and right 12 N sequence the corresponding coupled 12 N sequences were extracted and analyzed using the motif discovery tool MEME [33] (Additional file 1: Figure S8). Finally, the SynEs were analyzed *in silico* for the presence or absence of known plant DNA binding motifs by searching (Signal Scan) the PLACE database [34]. These analyses revealed that the isolated SynEs contained *bonafide* functional DNA motifs (e.g. W-box, GCC-box, *as-1* element) known to mediate transcriptional responses upon phytopathogen challenge [35,36]. However, many SynEs had no counterparts in the database suggesting that they were novel (Additional files 2 and 3).

**Figure 2:**
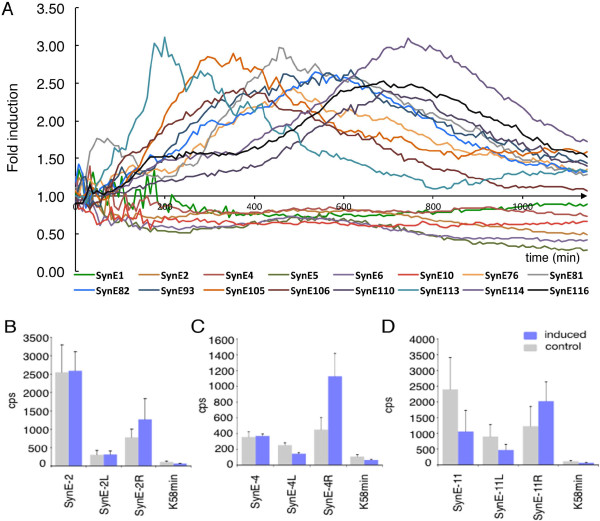
**Expression mediated by selected SynEs upon Pep25 treatment of parsley protoplasts. (A)** LUC activity was measured as counts per second (cps) of photon emission and Pep25-dependent fold induction values for the individual indicated SynEs, were calculated by dividing the counts per second (cps) values of the samples derived from stimulated protoplasts (induced state) with the corresponding cps values of the samples from untreated protoplasts for each time point. A subset of SynEs show inducibility upon Pep25 stimulation (ratio >1), whereas a subset of SynEs show Pep25-dependent decrease in activity (ratio <1) (see Additional file 7 for details). **(B**-**D)** LUC activities of three selected SynEs and their derivatives 4 hours after transfection into parsley protoplasts in the presence (induced) or absence (control) of Pep25 as indicated. SynE-2- (B, tcc-GACCTAGGTTGA-gaa(x)_14_atg-GCACAAGTTTGG-act), SynE-4- (C, tcc-ATTGAGACATAC-gaa(x)_14_atg-GCAGGACATTTG-act), and SynE-11- (D, tcc-ACCTGGGTGAAT-gaa(x)_14_atg-CTCTGTGCCTAG-act) mediated expressions were compared with those of derivatives containing only the 12 N-left (SynEs-L) or the 12 N-right(SynEs-R) sequence of the corresponding original SynEs, respectively. Note that SynE-2R, SynE-4R and SynE-11R supported enhanced transcriptional activity upon Pep25 stimulation. The transcriptional activity was measured as counts per second (cps) of photon emission produced by the LUC activity. The empty expression vector K58 min served as a control for background activity. A minimum of three biological replicates were performed for each SynE (error bars indicate SE).

Several putative Pep25-responsive SynCREs (Additional file 7) were selected for functional validation in parsley protoplasts and in stably transformed *Arabidopsis* plants. All tested SynEs supported transient reporter gene activity at a higher level compared to the empty vector control in the protoplasts but with varying strength. Eleven SynEs showed enhanced transcriptional activity upon Pep25 treatment (Figure 2a). Other frequently occurring SynEs (SynE1 to SynE11) showed higher transcriptional activity under non-inducing conditions indicating that they contain elements that negatively affect Pep25-dependent expression (Figure 2; Additional file 7). The limited number of individual tested Pep25 responsive elements enhanced expression by 2.2 to 3.1 fold. Although quite moderate, these results are consistent with previous reports in the animal field using defined cells or cell lines and similar plasmid reporter vectors [37,38]. Overall the sum of SynEs mediating Pep25 repression in the initial library may have been larger than those allowing activation. Activation of plant immune responses by elicitors such as Pep25 or flg22 have been shown to negatively impact on the expression of genes involved e.g. in photosynthesis, plant growth, DNA replication, auxin signaling, UV-B stress signaling and anthocyanin biosynthesis [39-42]. DNA elements known to mediate such responses were also identified in our SynE collection. Additionally, promoter-proximal pausing of RNA polymerase II, a recently revealed common feature in metazoans but not yet investigated in plants, may also have allowed capture of non-responsive SynEs due to Pol II recruitment [43]. Furthermore, several of the selected putative SynCREs may actually be composites containing distinct CREs that exert partly contrasting functions, constitutive/repressible or inducible, embedded within the two 12 N sites. To test this hypothesis, two additional versions of SynE1 to SynE11 were constructed to uncouple the 12 N-left (SynE-#L) from the 12 N-right (SynE-#R) sequence (Additional file 1: Figures S2, S9). Transcriptional activities of these versions revealed that three, SynE-2R, SynE-4R, and SynE-11R, mediated inducibility upon Pep25 treatment in the protoplasts (Figure 2b-d). SynE-4R showed the highest level of Pep25 inducibility (Additional file 1: Figure S10). Increasing the copy number of CREs can enhance inducible gene expression [2,44,45]. Thus, tandem versions of SynE-2, SynE-4 and SynE-11 were generated and tested in the transient protoplast assay. SynE-4 in diverse forward and reverse tandem combinations allowed a further increase in Pep25 inducibility (Additional file 1: Figure S11), whereas no further increase was observed with tandem versions of SynE-2 and SynE-11 (data not shown).

**Figure 3:**
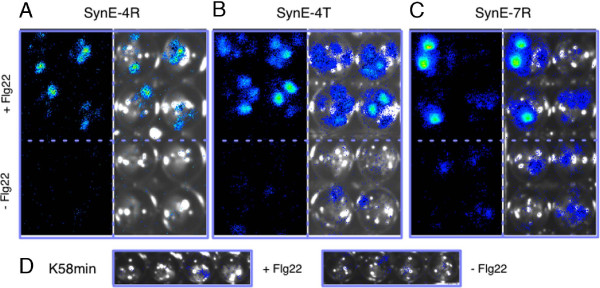
**Flg22-dependent *in-planta *transcriptional activities mediated by SynEs.** Flg22-responsiveness of two-week old *Arabidopsis* transgenic seedlings harboring the indicated SynE-4R (atg-GCAGGACATTTG-act), SynE-4R T (trimer of SynE-4R) and SynE-7R (atg-TGCTGACATAAA-act) expression constructs. Luciferin was added to the untreated (-Flg22) or treated (+Flg22) seedlings grown in micro-titer wells in liquid media and luciferase activity recorded using a CCD camera. The panels **(A)**, **(B)** and **(C)** show two images for each SynE reporter construct: one dark field image and the other merged with the bright field image. **(D)** A transgenic line carrying the empty expression vector (K58 min) used as control. Images were taken at 4 hours after treatment with flg22.

**Figure 4:**
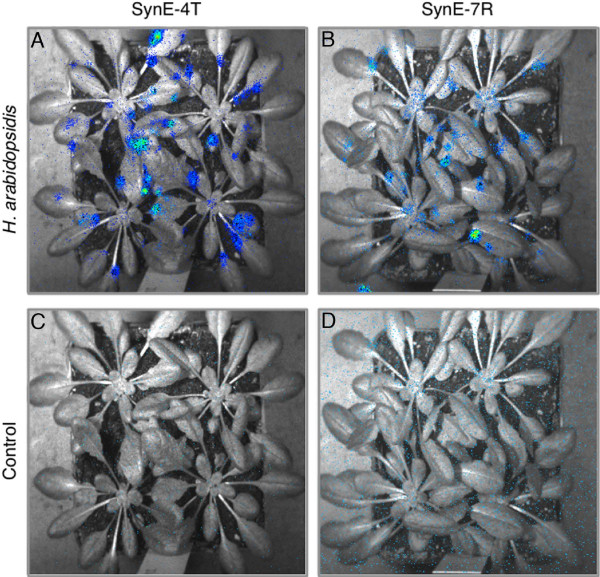
**Identified SynCREs can mediate pathogen-responsive expression in mature Arabidopsis plants.** Five week-old *Arabidopsis* transgenic plants carrying the indicated SynE expression constructs were challenged with the oomycete *Hyaloperonospora arabidopsidis* isolate Emwa **(A** and **B)** and expression of the *LUC* reporter gene was monitored and compared to unchallenged control plants **(C** and **D)**. Images were taken 6 hours upon application of *H. arabidopsidis* spores. The temporal dynamics of LUC activity under *H. arabidopsidis* challenge can be viewed in Additional files 9 and 10.

For *in planta* validation we transformed *Arabidopsis* to generate lines harboring SynCRE reporter constructs that were Pep25 responsive in the transient assay or that formerly showed little or no inducibility (Additional file 8). A previous study on semi-synthetic CREs has shown that the transcriptional regulation exerted *in planta* by such elements was tighter compared to the protoplast system [2]. Several independent transgenic lines were selected for each SynCRE construct and the transgene copy number determined (Additional file 8). Two-week old seedlings of T3 generation lines were treated with flg22, a bioactive 22 amino acid peptide from bacterial flagellin [46]. As demonstrated in Figure 3 flg22-dependent luciferase activity was detected in plantlets harboring selected SynCRE constructs. Similarly, SynCRE-mediated expression of the luciferase reporter construct was observed in five-week old T3 transgenic Arabidopsis lines challenged with spores of the oomycete *Hyaloperonospora arabidopsidis* (Figure 4). Temporal activation of the LUC reporter gene upon *H. arabidopsidis* challenge could clearly be observed as documented by the additional movie files (Additional files 9 and 10). In total four tested SynCREs were capable of supporting transcriptional activity mediated by flg22 and upon pathogen infection illustrating that our approach has succeeded in identifying such elements. The remaining lines showed no LUC activity upon stimulation. This may in part be due to co-suppression of the transgene in multi-copy lines, position effects at specific chromosomal sites, or non-responsiveness of such elements in leaf tissue, a situation reminiscent of the strong expression bias observed for some elements between different human cell lines [14].

## Conclusions

Our ability to identify functional SynCREs in plant cells illustrates the power of this method considering the numerous inherent difficulties associated with using plant material for such studies as compared to well-established animal systems. This methodology should reveal both previously unrecognized native DNA elements mediating transcriptional responses as well as synthetic elements whose strength and specificity may be highly suited to design tunable promoters for biotechnological approaches and for the development of artificial genetic systems employing diverse tissues and cell types. A key aspect of the described approach is that it can be applied to any transformable eukaryotic system to isolate and identify SynCREs active in transcription and/or responses to various environmental cues.

## Methods

### Generation of double stranded synthetic elements

The two randomized oligonucleotides were synthesized and generously provided by SIGMA-Genosys. For the randomized 12 N core a mix of dATP, dTTP, dCTP and dGTP in a ratio 1:1:1:1 was used during synthesis. The first oligonucleotide of 52 mers length was designed to contain a 5′ *Hind*III restriction endonuclease site, a 12 N core of random bases followed by a 20 mer sequence; the latter was present in reverse and complement fashion at the 3′ end of the second oligonucleotide (52 mer), which carried a 5′ *Asc*I restriction endonuclease site followed by a 12 N core of random bases (Additional file 1: Figure S2). Equimolar amounts of the two oligonucleotides (500 pmoles each) were mixed together. 1 μl of this oligo-mix was diluted to a final volume of 10 μl with 3× buffer (30 mM Tris–HCl pH 7.5, 150 mM NaCl, 30 mM MgCl2, 15 mM DTT, and 0.1 mg/ml gelatine) [47]. The oligo-mix was heated to 90°C for 3 minutes in a heating block. The heating block was subsequently switched off and the two oligonucleotides left to slowly anneal. After annealing the oligo-mix was subjected to primer extension adding 2.5 Units of Klenow fragment polymerase, 0.5 μl of dNTPs (20 mM) in a final volume of 30 μl, to obtain the randomized double stranded synthetic elements (designated SynEs). Finally, the randomized SynEs were NH_4_Ac/ ethanol precipitated, washed with 80% ethanol and resuspended to a final concentration of 100 ng/μl.

The two derivatives of a SynE, SynE-#L and SynE-#R, consisted of 3 tandem copies of each 12 N core and were generated by annealing two complementary oligonucleotides of 61 bases in length. The spacer of these derivatives was 6 base pair long (Additional file 1: Figure S2). A third derivative, SynE-#T, consisted of a tandem version of a SynE and was constructed by initially annealing two complementary oligonucleotides. The complementary oligonucleotides were designed to have a 10 base spacer sequence (Additional file 1: Figure S2). In addition, the double stranded SynE-#T had at its 5′ and 3′ ends a *Mlu*I and *Asc*I restriction endonuclease site overhang respectively, with a phosphate group at the corresponding 5′ ends. After annealing, the SynE-#T were ligated to obtain concatemers. Concatemer formation could occur by ligation of two *Mlu*I restriction sites, two *Asc*I restriction sites and/or the *Mlu*I*-Asc*I restriction sites, with the latter leading to disruption of the *Mlu*I and *Asc*I restriction sites. The ligation products were subsequently digested with *Asc*I and *Mlu*I thereby leaving only the concatemers ligated via *Mlu*I*-Asc*I restriction sites unaltered. The restriction digestion was followed by agarose gel electrophoresis to separate the various concatemers from the monomers. Concatemers of the desirable sizes were cut from the gel and the DNA extracted. The concatemers were ligated into the appropriate vector digested with *Asc*I and dephosphorylated with 10 units of *Alkaline Phosphatase*. After *E. coli* transformation with the ligation products, recombinant clones were selected by colony PCR. Dimers, trimers and tetramers were selected by gel electrophoresis and used for further studies.

### Library construction

One nanogram of the SynEs was subjected to 20 cycles PCR using High Fidelity Taq polymerase (ROCHE). The amplified dsSynEs were purified and restriction digested first with *Hind*III and subsequently with *Asc*I by adjusting the buffer conditions. Similarly, 10 μg a vector containing a multiple cloning site followed by a *35S CaMV* minimal promoter [48] and the intronless *LUCIFERASE* (*LUC*) reporter gene, was digested first with *Hind*III and purified on a sucrose gradient to isolate the fraction yielding linear digested vector DNA. The linearized DNA was then digested with *Asc*I followed by a second sucrose gradient to obtain highly purified vector DNA for cloning of the SynE fragments. Ligation reactions were performed using 1:2 equimolar ratio vector:SynEs in a volume reaction of 25 μl with a DNA concentration of about 5 ng/μl. Appropriate control ligation was performed to estimate background levels of non-recombinant vector molecules. 1–2 μl of the ligation reaction was used to transform highly electro competent *E. coli* cells (Invitrogen). A dilution series of the transformed *E. coli* cells was plated on LB medium with appropriate antibiotic to calculate the titer of the library. The rest of the transformed *E. coli* cells were store at 4°C overnight. Subsequently, a 25 ml liquid LB medium with antibiotic was inoculated with about 1x10^6^ of recombinant *E. coli* cells. This inoculum served as a pre-culture for inoculation of 500 ml LB medium to amplify the library and to yield sufficient plasmid DNA to perform parsley protoplast transient transfection assays.

### Transient protoplast luciferase assay

Parsley cells 5 days after sub-culturing were used for protoplast generation as previously described [49] with minor modifications. Protoplast transformation was performed by mixing 5 μg of linearized plasmid DNA, 200 μl of polyethylene glycol solution (25% polyethylene glycol, 100 mM Ca(NO_3_)_2_, 45 mM Mannit, pH 9.0) and 200 μl of protoplasts (about 1x10^6^ cells). After 30 minutes the protoplasts were washed once with 5 ml Ca(NO_3_)_2_ solution (275 mM Ca(NO_3_)_2_ 2 mM MES, pH 6.0), centrifuged at 1500 rpm and resuspended in 2 ml B5-sucrose solution (0,4 M sucrose, B5-salts and 5 μg/ml of 2,4-dichlorophenoxyacetic acid). Aliquots of 200 μl of the resuspended protoplasts were dispensed in a 96 well microtiter plate. 10 μl 5 mM luciferin was added to each well containing protoplasts, and Pep25 solution (final concentration of 2 μg/ml) was added to half of the wells, whereas the other half served as controls. Plates were sealed with parafilm. One or multiple plates were loaded on a Top-Count device (Perkin-Elmer) to measure photon emission at time intervals of 3 minutes. The photon emission, recorded by the Top-Count, is the result of the LUCIFERASE activity and is a direct measure of the transcriptional activity of the *LUCIFERASE* gene, which in turn depends on the ability of the SynEs to mediate expression. Monitoring of photon emission over time for each selected SynE or for a transformed library of SynEs permitted to follow the temporal transcriptional activity supported by the SynE(s) under investigation.

### Generation of transgenic plants

All SynE derivatives were cloned into the binary vector K58min upstream of the *35S CaMV* minimal promoter:*LUCIFERASE* reporter gene cassette and electroporated into *Agrobacterium tumefaciens* strain GV3101pMP90RK. All constructs were transformed into Arabidopsis Col-0 plants using the floral dipping method [50]. Transformants were selected on MS agar medium containing 15 g/ml of DL-phosphinothricin.

### Plant growth and treatments

Transgenic Arabidopsis plants were grown in chambers (SNIJDER B.V) under a 12 h light/dark cycle regime at 23°C and 60% humidity. Five-week old plants were spray-inoculated with spores (4 × 10^4^ spore ml^-1^) of the oomycete *H. arabidopsidis* isolate Emwa. For flg22 treatments Arabidopsis seedlings were grown in macrotiter wells (black clear bottom VisiPlate-24; Perkin Elmer) containing 1 ml MS medium with reduced sucrose (2.5 g L^-1^) under a 12 h light/dark cycle at 21°C and 70% humidity. Addition of flg22 (50 μl of 22 μM flg22 per well) to the medium of two-week old seedling was for 2 hours.

### Photon imaging

All photon imaging of plants carrying the luciferase reporter constructs for the *in planta* experiments were recorded using a photon counting camera by HAMAMATSU model C2400-40H. The camera was set to an exposure time of 1 h and left running for 12-72 h.

### Sequence pre-processing

Thirty six nucleotide long paired-end Illumina sequences were obtained as fasta files. Barcode tags were used to group each of the sequences to the eight different samples (Additional file 1: Figure S5; Additional file 11). Each forward and reverse sequence spanned the left and the right 12 N randomized cores of a SynE, respectively. The reverse sequences were first reverse-complemented and then concatenated with the corresponding forward sequence by mean of a sequence identifier common to the two mate pair sequences. SynEs sequences were extracted using the experimentally designed SynE pattern based solely on position, which included the last 18 nucleotides for the left 12 N and the first 18 nucleotides for the right 12 N. To exclude sequences including errors caused by biochemical and/or by sequencing processing, the sequences were additionally checked for the presence of the flanking nucleotides. Those sequences matching the regular expression TCC[ACGT]{12}GAA for the left core region and ATG[ACGT]{12}ACT for the right core region respectively, were extracted as bona fide SynEs. Not included were sequences containing ambiguous nucleotides (Ns) at any of the random 12 core positions. Moreover, we “fixed” the flanking sequences TCC … GAA and ATG … ACT to avoid possible shifts in the sequence. All sequence pre-processing steps were implemented in Perl-scripts.

### Formaldehyde cross-linking and chromatin immunoprecipitation

Cross-linking of parsley protoplasts were performed three hours post transformation. At this time point luciferase activity has already increased but did not reach its maximum. Cross-linking was performed by adding formaldehyde to the final concentration of 1% to the microtiter wells containing the transformed protoplasts for 5 minutes. After cross-linking, the excess of formaldehyde was quenched by adding glycine to 0.125 M final concentration. 2 ml protoplasts were transferred with a pipette into 15 ml tubes, the volume adjusted to 7 ml with CaCl_2_, and subsequently gently pelleted by centrifugation at 1500 rpm for 5 minutes. Soluble chromatin was extracted by adding 300 μl of sonication buffer (10 mM Tris–HCl, pH 7.5, 1 mM EDTA pH 8.0, 0.5 mM EGTA, 10 mM Na Pyrophosphate, 0.5% SDS) [20] to the pellet. The soluble cross-linked chromatin was sheared using a Bioruptor™ sonicator for a total of 5 min with 20s continuous pulses and 60s interruption periods, with instrumentation settings at low power. After diluting the chromatin to 0.1% SDS, chromatin-Pol-II complexes were immunoprecipitated using the H14 antibody (Covance) specific to the Ser-5 phosphorylated form of Pol-II. H14 was immobilized on magnetic beads as previously described [20,51]. Following immunoprecipitation and washing [20] the chromatin immunoprecipitated (ChIPed) fragments were released from proteins by proteinase K digestion followed by an RNase digestion, and the cross-link reversed overnight at 65°C. The ChIPed fragments were extracted once with phenol/chloroform/isoamyl alcohol, once with chloroform, precipitated with NaAc/ethanol and washed once with 80% ethanol. The ChIPed fragments were resuspended in 50 μl TE buffer, pH 8.0. A 1:5 dilution of the ChIPed fragments were used in a PCR reaction to specifically amplify the cassette containing the tandem 12 N sequences. This PCR product served as starting material to construct a second SynE library for a further round of RNApol-II ChIP. Alternatively, the ChIPed fragments were amplified with specific primers carrying a bar-coded flag-sequence required for paired-end Solexa sequencing.

### Clustering

We computed a non-redundant sequence set for each sample, and counted the occurrence number of each sequence (Table 1). The non-redundant sequences with their assigned frequencies were clustered using the program FreClu [32]. Originally, this method was developed to cluster short sequences representing the same genomic region of a reference genome but mapping to a different position due to sequencing errors. If two sequences differ only in one nucleotide (Hamming distance = 1) a statistical test based on the frequency and on the base quality scores, decides if a sequencing error causes a mismatch or if the sequences belong two different loci. With this clustering method, each cluster is represented by a parent sequence, with the highest frequency value, and by a cumulative frequency, which is the sum of frequencies of all members in the cluster. For our purpose, we allowed clustering only based on the frequencies and on the Hamming distance. Therefore we modified the input file for the FreClu program, setting all base quality scores of our SynEs sequences to the low Phred Score of 2. Further analysis or comparisons of the cluster composition amongst samples were based on representative sequences.

### Motif discovery

Several approaches were used to identify new and known motifs present on the non-redundant sequence data. Permutation sets of all possible five to seven nucleotides long motifs were created and the number of their occurrences in each sample was counted. Frequency ratios for the motifs in the immunoprecipitated samples and in the libraries were calculated to reveal overrepresented/enriched motifs. In a second approach, the command line version of motif discovery tool MEME (MEME 4.3) [33] was used to search for significant motifs in a subset of the non-redundant sequences. The left and right 12 N SynE cores were extracted from the most frequent sequences of the chromatin immunoprecipitated samples to retrieve all the corresponding right and left 12 N cores coupled with them. MEME searched for motifs in the variable 12 N core sequences. Finally, known motifs were identified in the representative sequences of the clusters. Therefore, the PLACE database [34] was downloaded and filtered from redundant sequences and motifs that were longer than 14 bases (original DB 469 entries, filtered DB 376 entries). The SignalScan [sigscan4] [52] program was downloaded  (http://genamics.com/software/downloads/sigscan405.shar) and installed locally to scan the PLACE database. All developed scripts to perform the bioinformatics analyses were implemented in Perl (perl v5.8.8).

## Competing interests

The authors declare that they have no conflict of interests.

## Authors’ contributions

MR carried out the design of the experiments, generated the randomized synthetic element libraries, performed the transformations and the activity assays, generated the transgenic plants, helped in the statistical analysis, and co-drafted the manuscript. NA and TC performed the statistical analyses and established the bioinformatics pipeline. IES was involved in the conception and design of the experiments and co-authored the manuscript. All authors read and approve the final manuscript.

## Supplementary Material

Additional file 1: Figure S1 Strategy used to construct a double stranded randomized synthetic elements (SynE). Figure S2 Molecular features of the double stranded randomized synthetic elements (SynE). Figure S3 Protein blots to validate the specificity of the H14 antibody used for detection of plant Ser-5 phosphorylated Pol II. Figure S4 *LUC* activity detected in parsley protoplasts. Figure S5 Barcoding of the SynEs for paired-end sequencing. Figure S6 Flow-chart of the bioinformatic approach developed to analyze the raw sequence data and to select candidate SynCREs. Figure S7 Venn-diagram representations of sequences within ChIP samples. Figure S8 Correlation of left and right 12 N sequence combinations.. Figure S9 Transient transcriptional activities of specific 12xN-left sequence and 12×N-right sequence reporter constructs detected in parsley protoplasts. Figure S10 Induced expression mediated by the three SynEs-#R upon Pep25 treatment of parsley protoplasts. Figure S11 Induced expression mediated by tandem versions of SynE-4 upon Pep25 treatment of parsley protoplasts. Click here for file

Additional file 2 Common representative enriched SynEs in sample libraries S4, S5 and S8. Click here for file

Additional file 3 Common representative enriched SynEs in sample libraries S6 and S7. Click here for file

Additional file 4 Frequencies of five nucleotide long permutations in the sub-libraries from which the immunoprecipitated sample originated. Click here for file

Additional file 5 Frequencies of six nucleotide long permutations in the sub-libraries from which the immunoprecipitated sample originated. Click here for file

Additional file 6 Frequencies of seven nucleotide long permutations in the sub-libraries from which the immunoprecipitated sample originated. Click here for file

Additional file 7 Pep25-responsiveness of selected candidate SynEs. Click here for file

Additional file 8 **List of putative SynEs used to generate transgenic *Arabidopsis thaliana *** **plants.** Click here for file

Additional file 9 **The temporal dynamics of the LUC activity of SynE-4 T Trimer forward under *H. arabidopsidis *** **challenge.** Arabidopsis plants harbouring the transgene were sprayed with 5 μM luciferin and placed under the photon counting camera for bright field imaging. To monitor background signal, the camera was set to an exposure time of 1 h and left running 2 h. Subsequently, several plants were spray-infected with the spores of *H. arabidopsidis* using an ECOSPRAY ATOMIZER. The infected plants were carefully returned (avoiding touching) along with control plants. The camera was again set to an exposure time of 1 h and left running for 12-72 h. Click here for file

Additional file 10 **The temporal dynamics of the LUC activity of SynE-7R under *H. arabidopsidis *** **challenge.** Arabidopsis plants harbouring the transgene were sprayed with 5 μM luciferin and placed under the photon counting camera for bright field imaging. To monitor background signal, the camera was set to an exposure time of 1 h and left running 2 h. Subsequently, several plants were spray-infected with the spores of *H. arabidopsidis* using an ECOSPRAY ATOMIZER. The infected plants were carefully returned (avoiding touching) along with control plants. The camera was again set to an exposure time of 1 h and left running for 12-72 h. Click here for file

Additional file 11 Primers used in this study. Click here for file
