# Supplementary figures and images for: Identification of functional cis-regulatory elements by sequential enrichment from a randomized synthetic DNA library
Source: BMC Plant Biol. 2013 Oct 18;13:164. doi: 10.1186/1471-2229-13-164 (PMC3923269; doi:10.1186/1471-2229-13-164)

## Slide 1
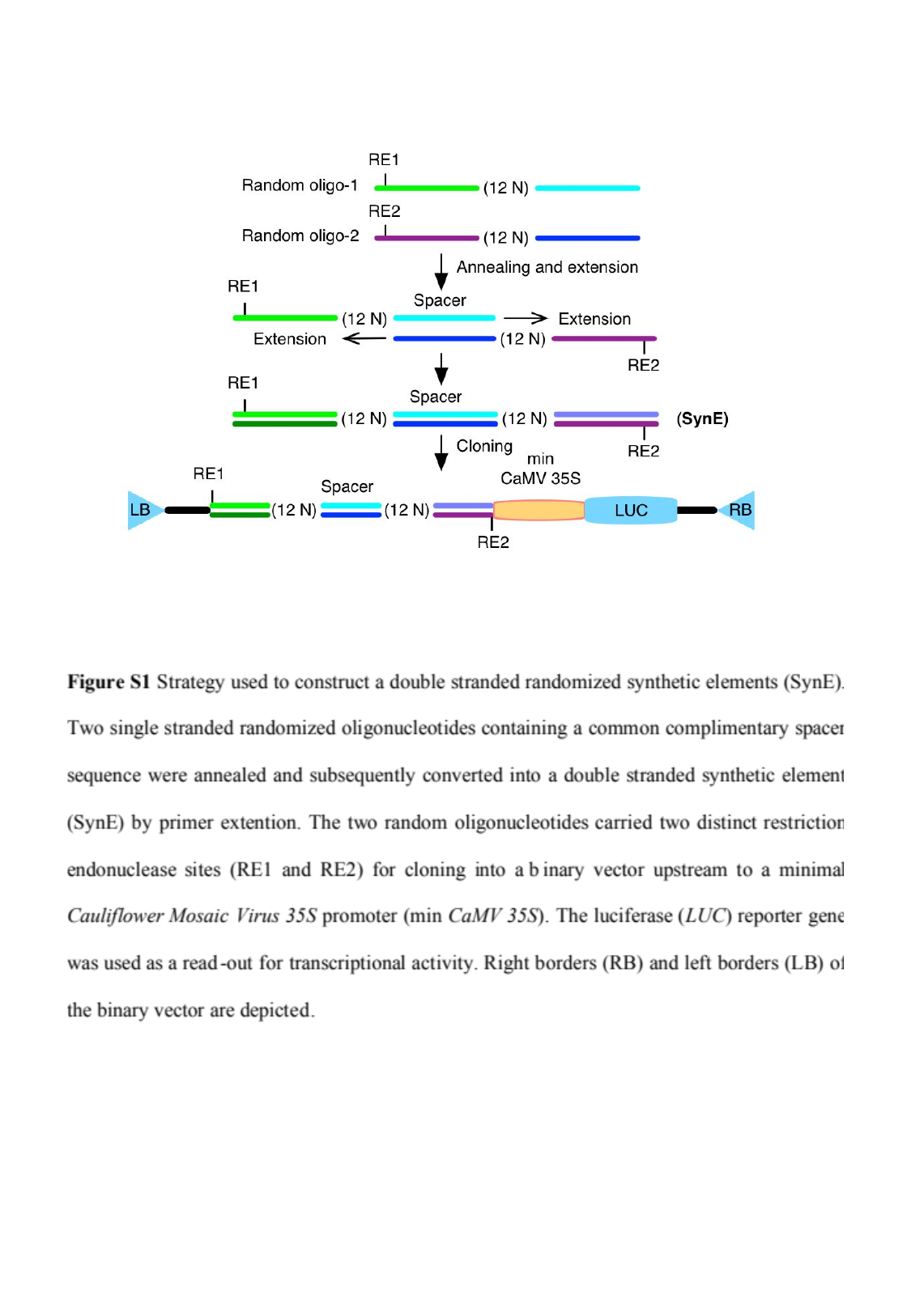

## Slide 2
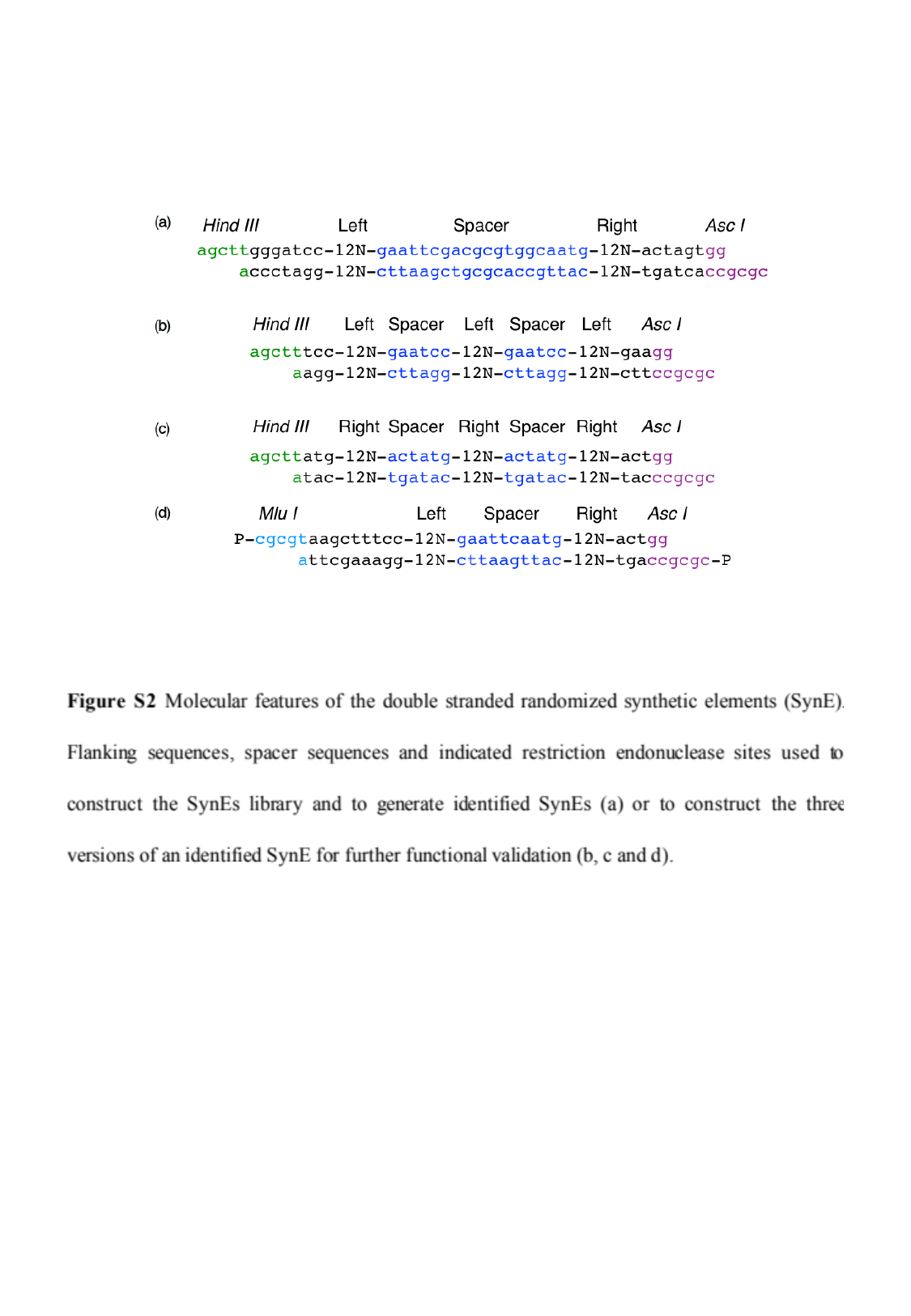

## Slide 3
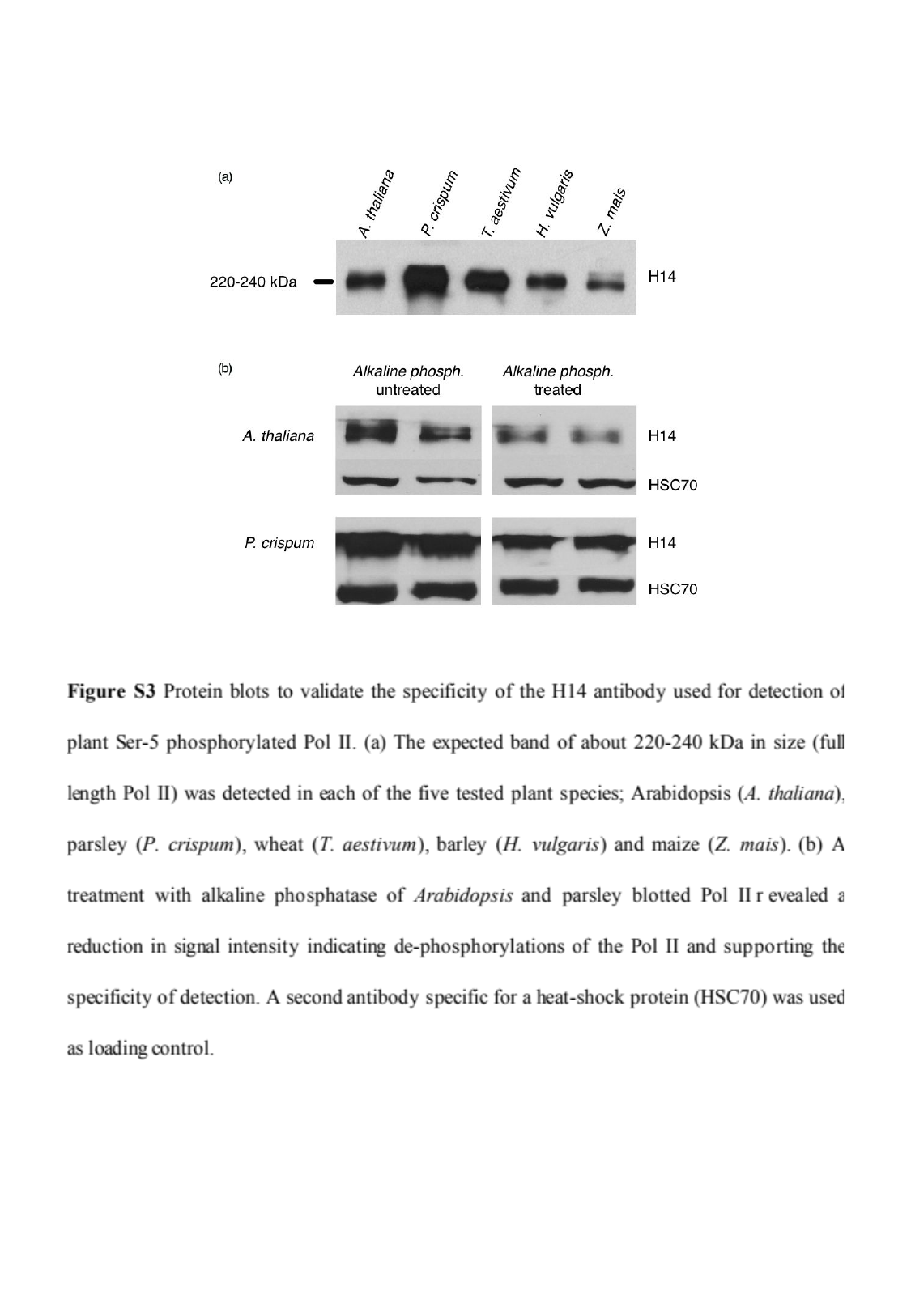

## Slide 4
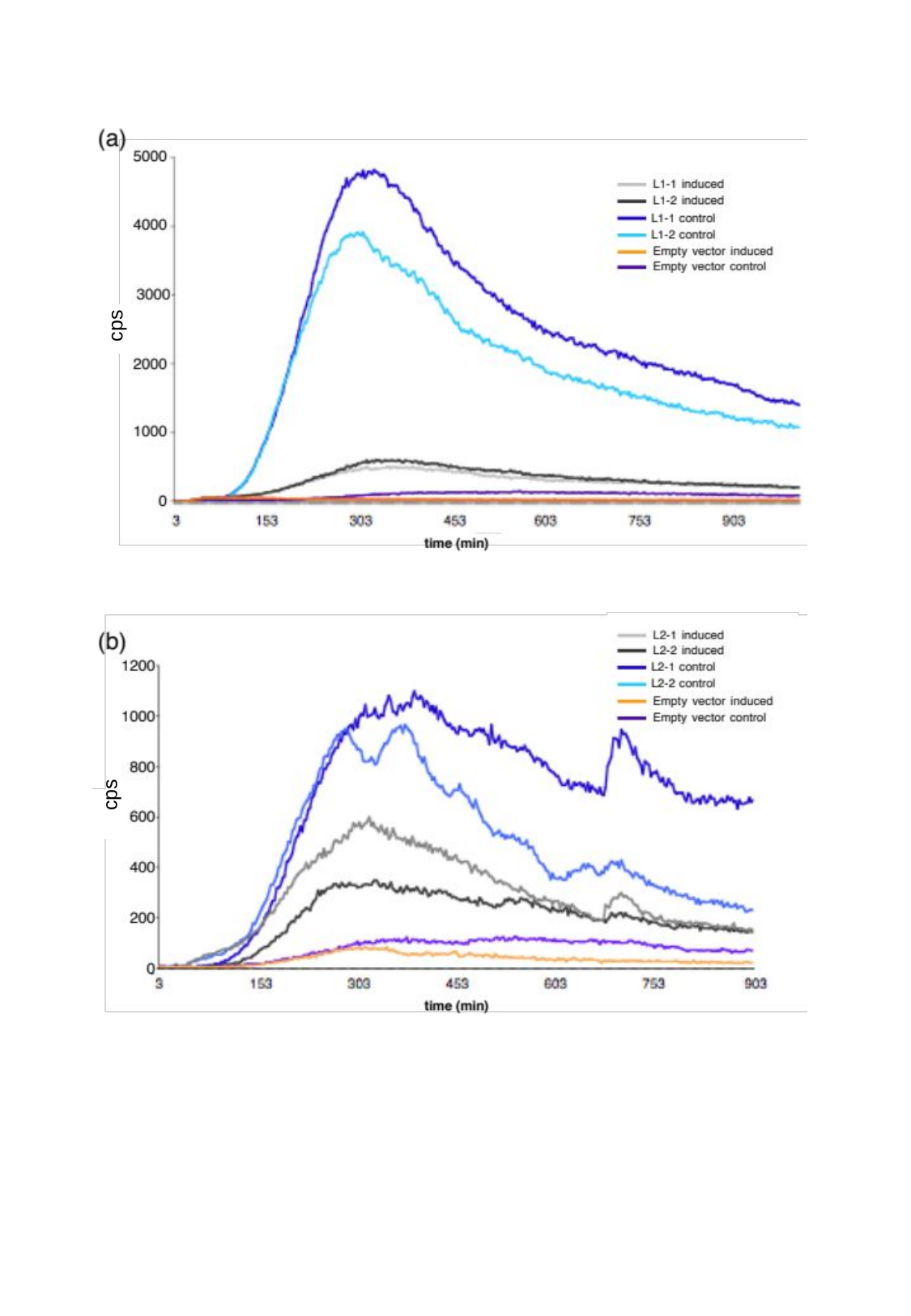

cps
cps

## Slide 5
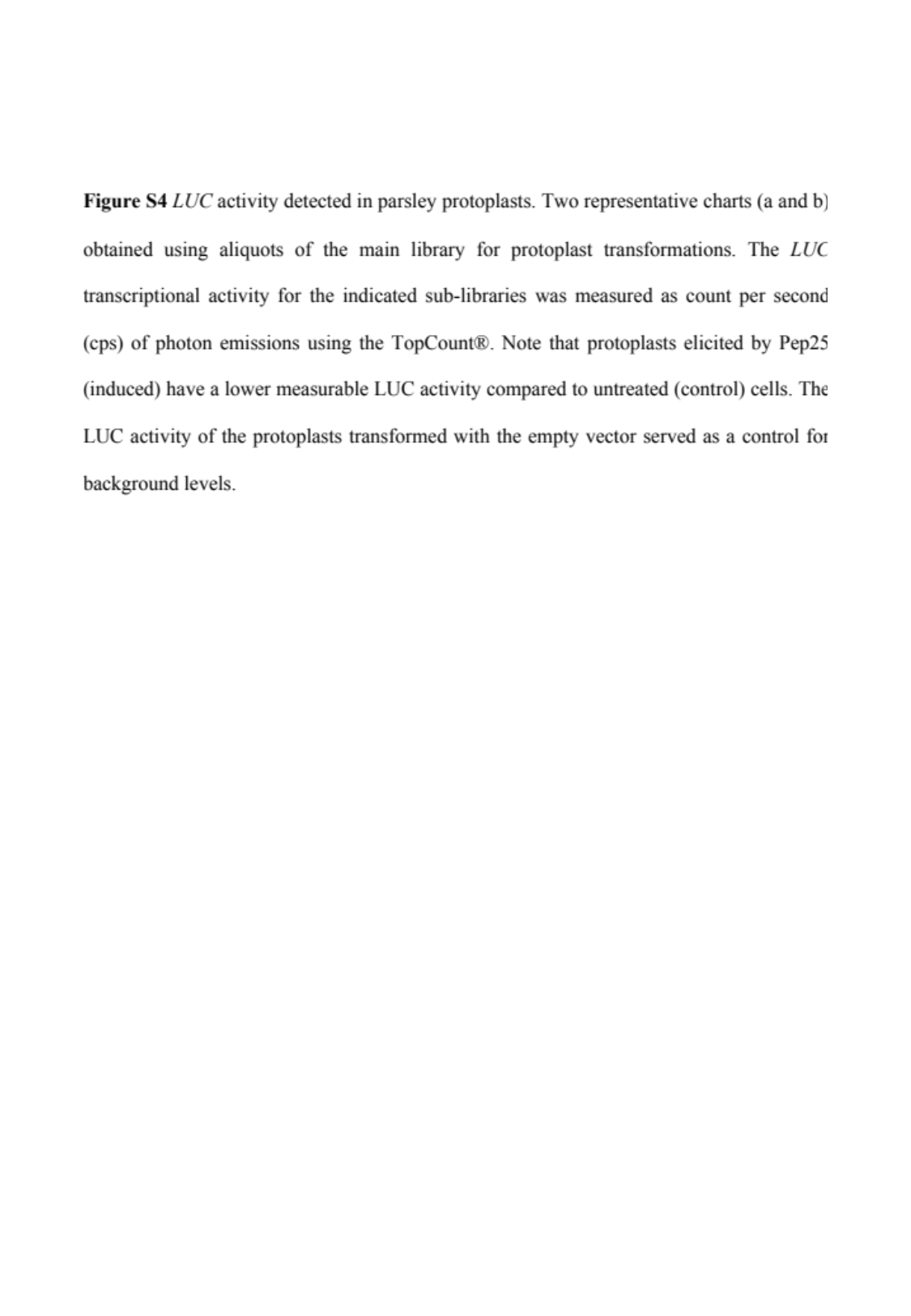

## Slide 6
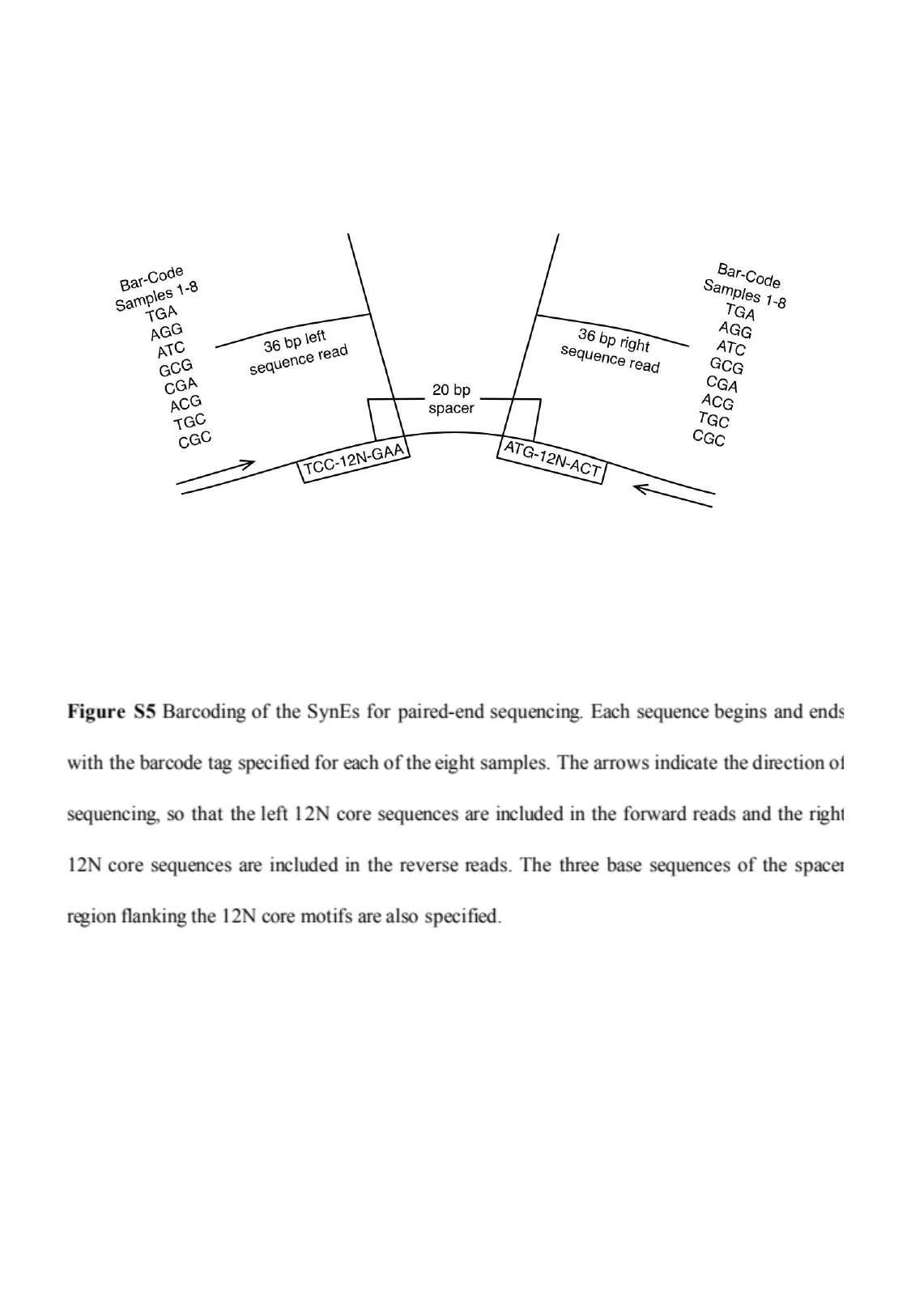

## Slide 7
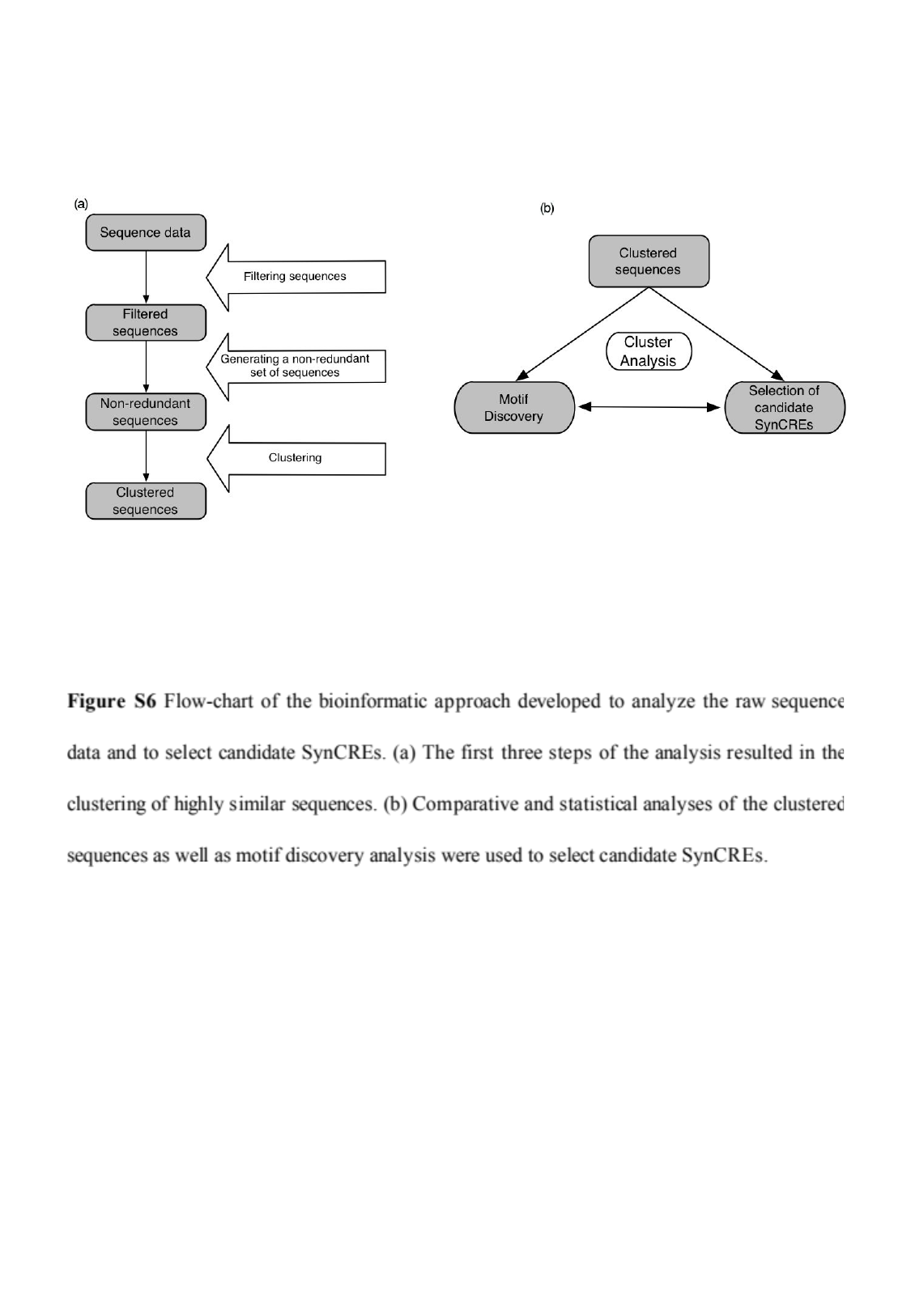

## Slide 8
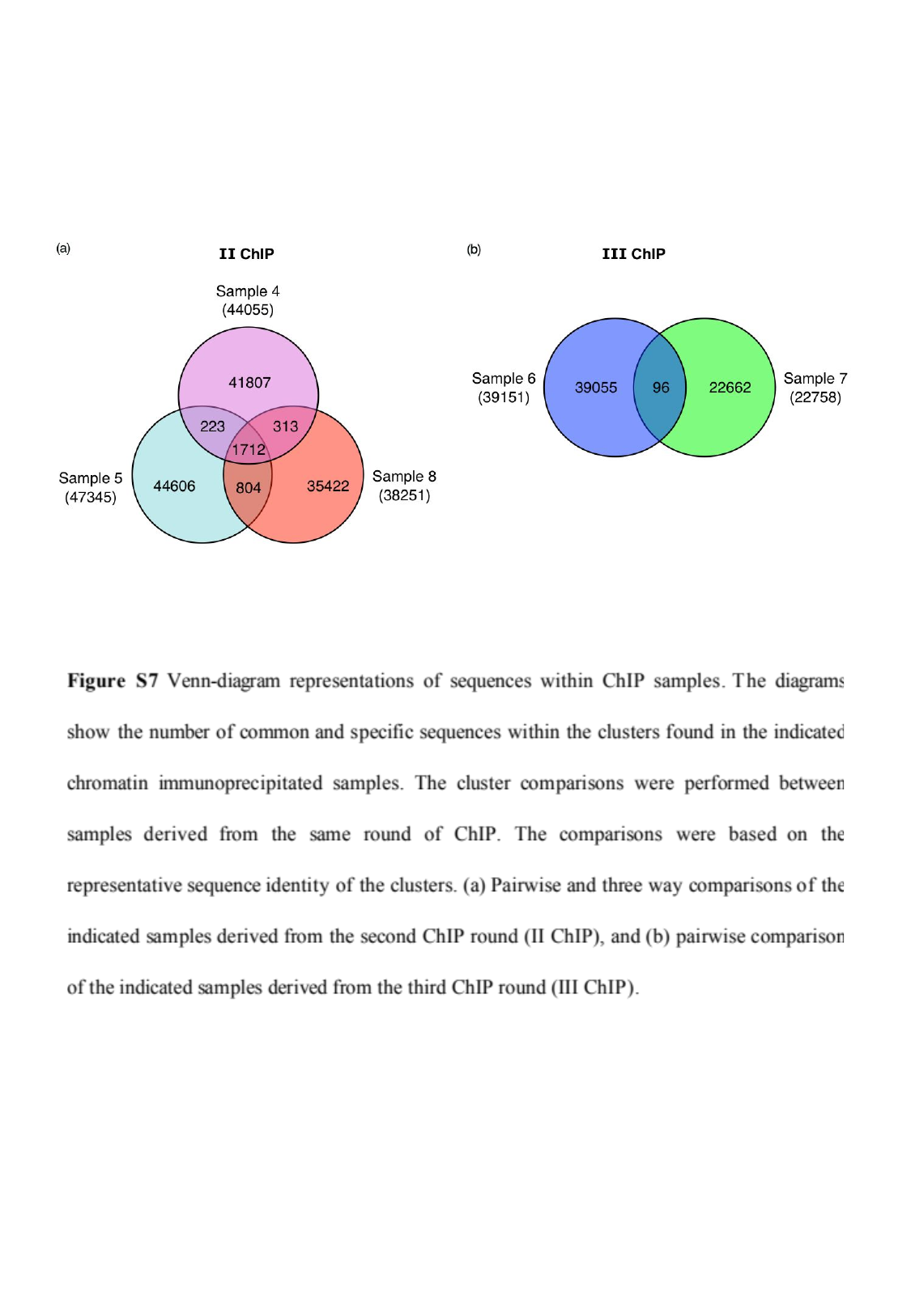

## Slide 9
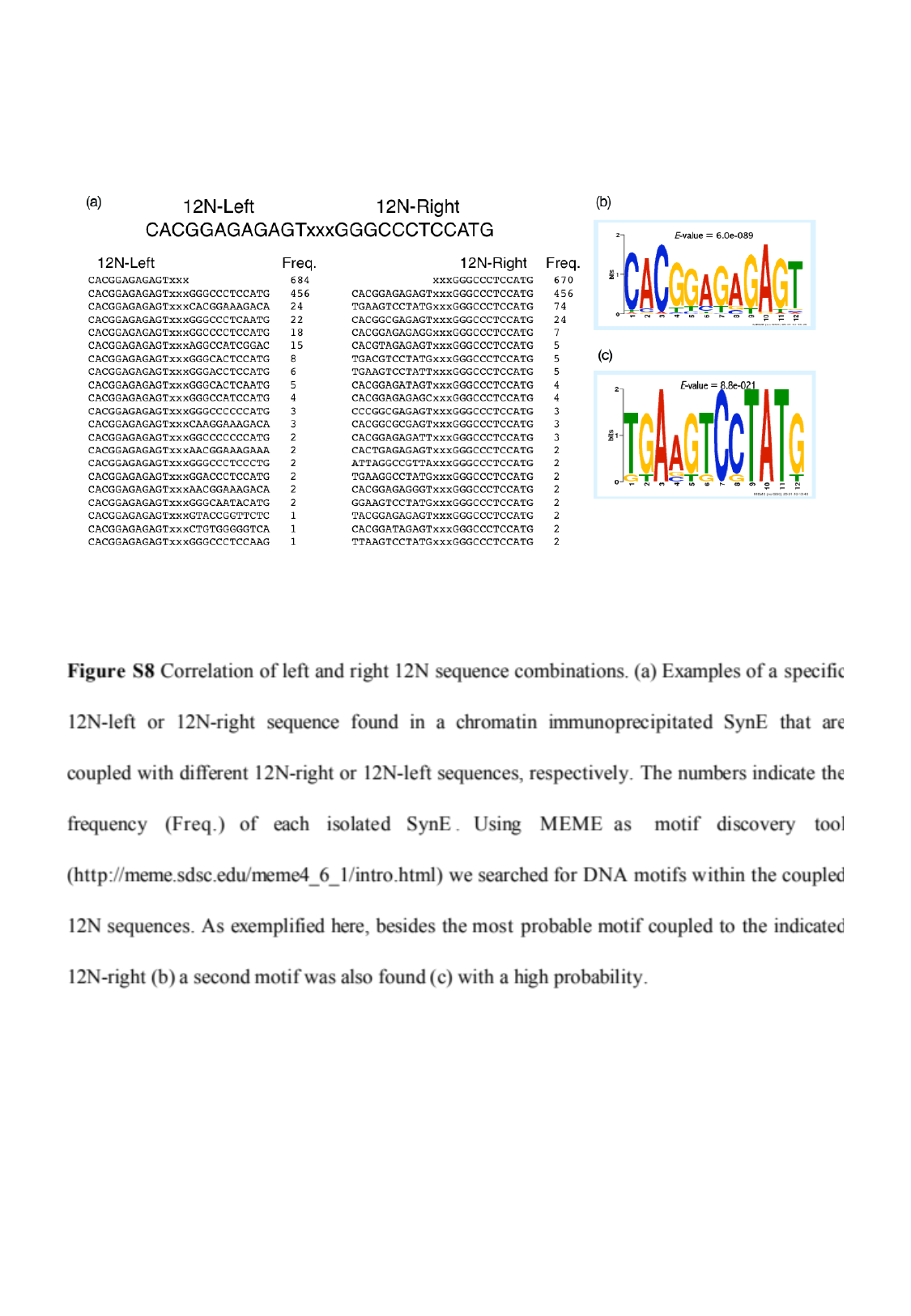

## Slide 10
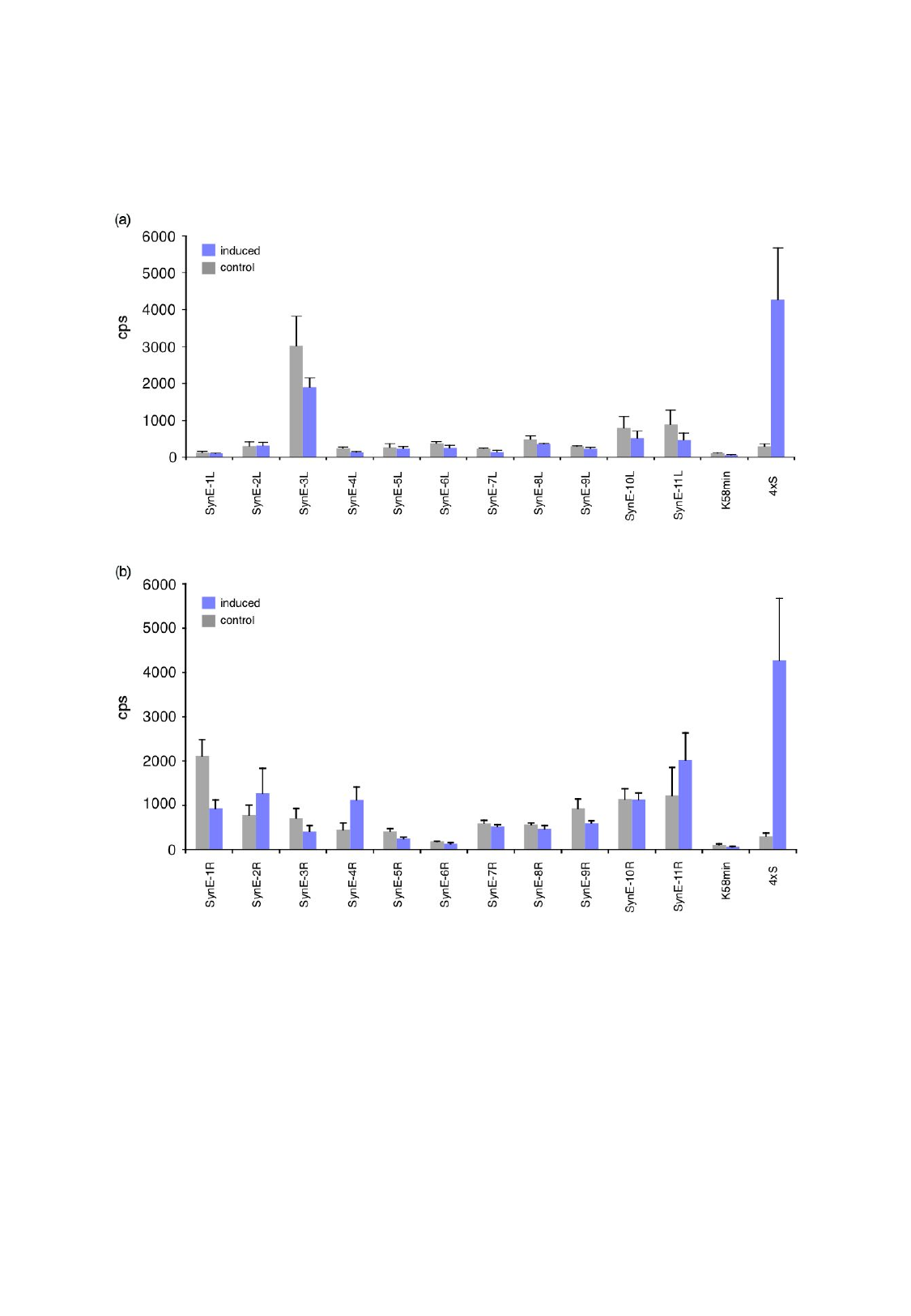

## Slide 11
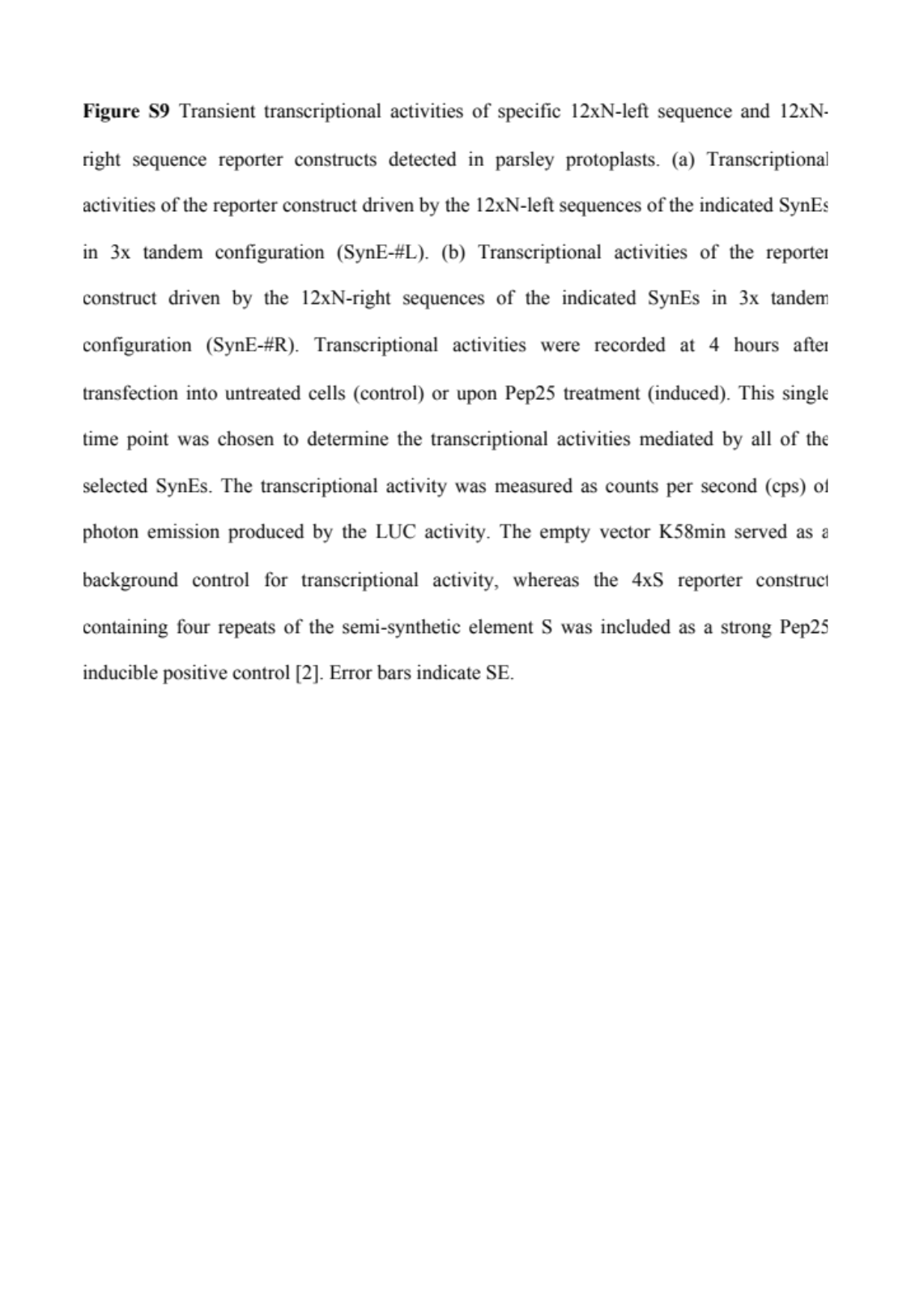

## Slide 12
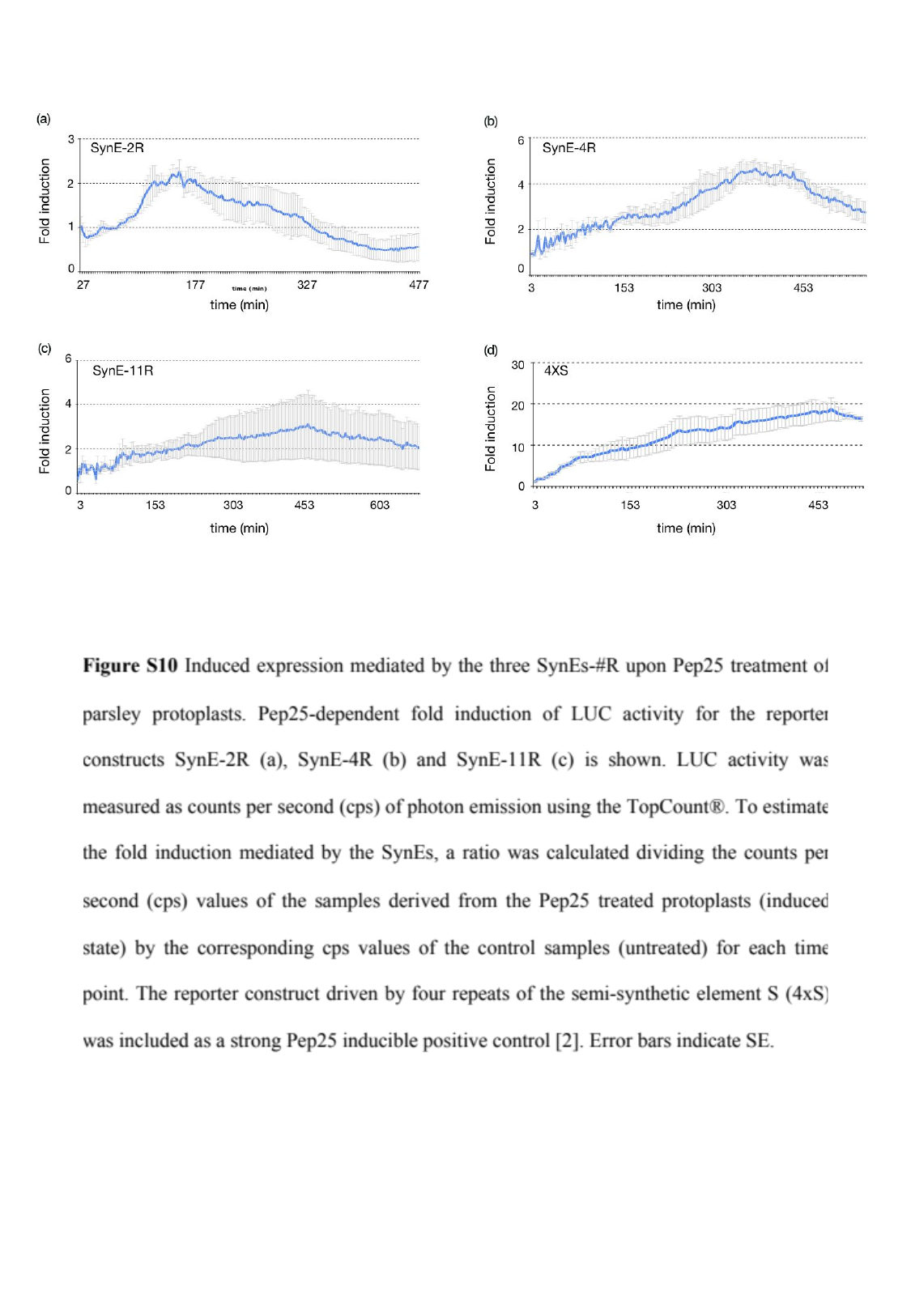

## Slide 13
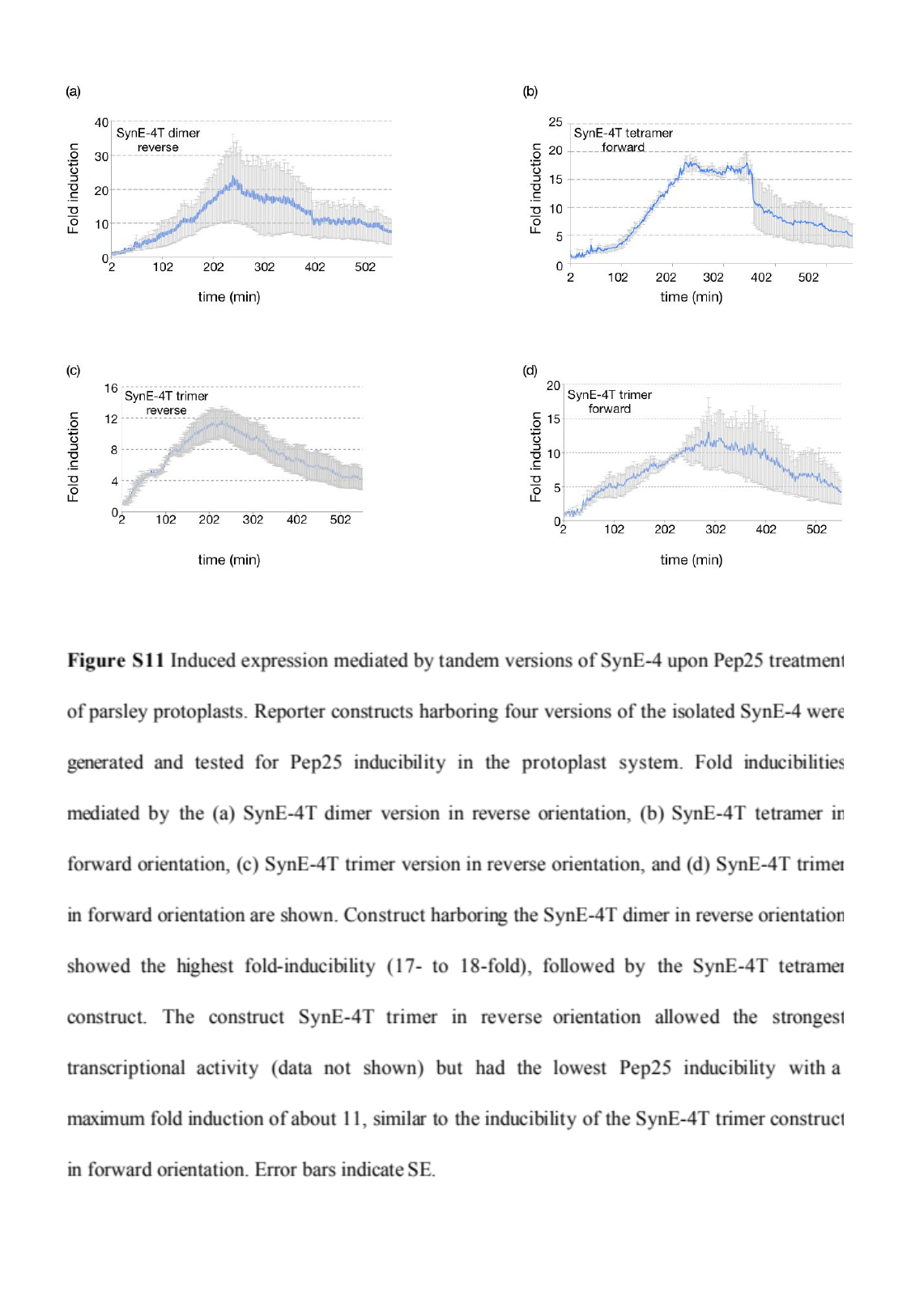

Supplement: Additional file 1: Figure S1 — Strategy used to construct a double stranded randomized synthetic elements (SynE). Figure S2 Molecular features of the double stranded randomized synthetic elements (SynE). Figure S3 Protein blots to validate the specificity of the H14 antibody used for detection of plant Ser-5 phosphorylated Pol II. Figure S4 LUC activity detected in parsley protoplasts. Figure S5 Barcoding of the SynEs for paired-end sequencing. Figure S6 Flow-chart of the bioinformatic approach developed to analyze the raw sequence data and to select candidate SynCREs. Figure S7 Venn-diagram representations of sequences within ChIP samples. Figure S8 Correlation of left and right 12 N sequence combinations.. Figure S9 Transient transcriptional activities of specific 12xN-left sequence and 12×N-right sequence reporter constructs detected in parsley protoplasts. Figure S10 Induced expression mediated by the three SynEs-#R upon Pep25 treatment of parsley protoplasts. Figure S11 Induced expression mediated by tandem versions of SynE-4 upon Pep25 treatment of parsley protoplasts. [file 1471-2229-13-164-S1.ppt]
