# Supplementary material for: Identification of functional cis-regulatory elements by sequential enrichment from a randomized synthetic DNA library
Source: BMC Plant Biol. 2013 Oct 18;13:164. doi: 10.1186/1471-2229-13-164 (PMC3923269; doi:10.1186/1471-2229-13-164)
Supplement: Additional file 11 — Primers used in this study. [file 1471-2229-13-164-S11.doc]

**List of primers used in this study**

| Primer Name | Sequence | Function |
| --- | --- | --- |
| Random3 | 5’-tgagcccaagcttgggatcc12Ngaattcgacgcgtggcaatg-3’ | Forward oligonucleotide containing a 12N left randomized core and a *Hind-III* restriction site for cloning. |
| Random4 | 5’-gctaaaggcgcgccactag12Ncattgccacgcgtcgaattc | Partial complementary oligonucleotide containing a 12N right randomized core and a *Asc-I* restriction site for cloning. |
| For. Random3 | 5’-tgagcccaagcttgggatcc-3’ | Forward oligonucleotide for the amplification of the synthetic randomized element cassette. |
| Rev. Random4 | 5’-gctaaaggcgcgccactagt-3’ | Reverse oligonucleotide for the amplification of the synthetic randomized element cassette. |
| Min35pr1 | 5’-cgcgtgcagcggatcaagcttgg-3’ | Forward oligonucleotide for the amplification of chromatin immunoprecipitated SynEs. |
| Luc Rev pr1 | 5’-ttggcgtcttccatggtggc-3’ | Reverse oligonucleotide for the amplification of chromatin immunoprecipitated SynEs. |
| BAR-Library 5’primer | 5’-tctttccctacacgacgctcttccgatcttgaagcccaagcttgggatcc-3’ | Forward oligonucleotide to bar-code the main library (sample 1) of randomized SynEs. Three mers bar-code is indicated in red. |
| BAR-Library 3’primer | 5’-ggcattcctgctgaaccgctcttccgatcttcataaaggcgcgccactagt-3’ | Reverse oligonucleotide to bar-code the main library (sample 1) of randomized SynEs. Three mers bar-code is indicated in red. |
| Bar-1 5’ primer | 5’-tctttccctacacgacgctcttccgatctaggggatcaagcttgggatcc-3’ | Forward oligonucleotide to bar-code the sub-library 1 (sample 2) of randomized SynEs. Three mers bar-code is indicated in red. |
| Bar-1 3’primer | 5’ ggcattcctgctgaaccgctcttccgatctccttagtggcgcgccactagt-3’ | Reverse oligonucleotide to bar-code the sub-library 1 (sample 2) of randomized SynEs. Three mers bar-code is indicated in red. |
| Bar-2 5’ primer | 5’- tctttccctacacgacgctcttccgatctatcggatcaagcttgggatcc-3’ | Forward oligonucleotide to bar-code the sub-library 2 (sample 3) of randomized SynEs. Three mers bar-code is indicated in red. |
| Bar-2 3’primer | 5’-ggcattcctgctgaaccgctcttccgatctgattagtggcgcgccactagt-3’ | Reverse oligonucleotide to bar-code the sub-library 2 (sample 3) of randomized SynEs. Three mers bar-code is indicated in red. |
| Bar-3 5’ primer | 5’-tctttccctacacgacgctcttccgatctgcgggatcaagcttgggatcc-3’ | Forward oligonucleotide to bar-code the chromatin immunoprecipitated sample 4. Three mers bar-code is indicated in red. |
| Bar-3 3’primer | 5’-ggcattcctgctgaaccgctcttccgatctcgctagtggcgcgccactagt-3’ | Reverse oligonucleotide to bar-code the chromatin immunoprecipitated sample 4. Three mers bar-code is indicated in red. |
| Bar-4 5’ primer | 5’-tctttccctacacgacgctcttccgatctcgaggatcaagcttgggatcc-3’ | Forward oligonucleotide to bar-code the chromatin immunoprecipitated sample 5. Three mers bar-code is indicated in red. |
| Bar-4 3’primer | 5’-ggcattcctgctgaaccgctcttccgatcttcgtagtggcgcgccactagt-3’ | Reverse oligonucleotide to bar-code the chromatin immunoprecipitated sample 5. Three mers bar-code is indicated in red. |
| Bar-5 5’ primer | 5’-tctttccctacacgacgctcttccgatctacgggatcaagcttgggatcc-3’ | Forward oligonucleotide to bar-code the chromatin immunoprecipitated sample 6. Three mers bar-code is indicated in red. |
| Bar-5 3’primer | 5’-ggcattcctgctgaaccgctcttccgatctcgttagtggcgcgccactagt-3’ | Reverse oligonucleotide to bar-code the chromatin immunoprecipitated sample 6. Three mers bar-code is indicated in red. |
| Bar-6 5’ primer | 5’-tctttccctacacgacgctcttccgatcttgcggatcaagcttgggatcc-3’ | Forward oligonucleotide to bar-code the chromatin immunoprecipitated sample 7. Three mers bar-code is indicated in red. |
| Bar-6 3’primer | 5’-ggcattcctgctgaaccgctcttccgatctgcatagtggcgcgccactagt-3’ | Reverse oligonucleotide to bar-code the chromatin immunoprecipitated sample 7. Three mers bar-code is indicated in red. |
| Bar-7 5’ primer | 5’-tctttccctacacgacgctcttccgatctcgcggatcaagcttgggatcc-3’ | Forward oligonucleotide to bar-code the chromatin immunoprecipitated sample 8. Three mers bar-code is indicated in red. |
| Bar-7 3’primer | 5’-ggcattcctgctgaaccgctcttccgatctgcgtagtggcgcgccactagt-3’ | Reverse oligonucleotide to bar-code the chromatin immunoprecipitated sample 8. Three mers bar-code is indicated in red. |
| Solexa 5’primer | 5’-aatgatacggcgaccaccgagatctacactctttccctacacgacgc-3’ | Forward primer to amplify libraries and chromatin immunoprecipitated samples for Solexa sequencing. |
| Solexa 3’primer | 5’-caagcagaagacggcatacgagatcggtctcggcattcctgctgaaccg-3’ | Reverse primer to amplify libraries and chromatin immunoprecipitated samples for Solexa sequencing. |
